# Light in the Senior Home: Effects of Dynamic and Individual Light Exposure on Sleep, Cognition, and Well-Being

**DOI:** 10.3390/clockssleep2040040

**Published:** 2020-12-14

**Authors:** Myriam Juda, Teresa Liu-Ambrose, Fabio Feldman, Cristian Suvagau, Ralph E. Mistlberger

**Affiliations:** 1 Sleep and Circadian Neuroscience Laboratory, Department of Psychology, Simon Fraser University, Burnaby, BC V5A 1S6, Canada; 2 Circadian Light Therapy, 149 W. 13th Ave., Vancouver, BC V5Y 1V8, Canada; 3 Aging, Mobility, and Cognitive Neuroscience Laboratory, Department of Physical Therapy, University of British Columbia, Vancouver, BC V6T 1Z3, Canada; teresa.ambrose@ubc.ca; 4 Djavad Mowafaghian Centre for Brain Health, University of British Columbia, Vancouver, BC V6T 1Z3, Canada; 5 Centre for Hip Health and Mobility, Vancouver Coastal Health Research Institute, Vancouver, BC V5Z 1M9, Canada; 6 Clinical Quality and Patient Safety, Fraser Health Authority, 13450 102nd Ave., Surrey, BC V3T 5X3, Canada; Fabio.Feldman@fraserhealth.ca; 7 Conservation and Energy Management Engineering, BC Hydro, 333 Dunsmuir St., Vancouver, BC V6B 5R3, Canada; cristian.suvagau@bchydro.com

**Keywords:** circadian rhythms, sleep, light, entrainment, cognition, aging, nursing home

## Abstract

Disrupted sleep is common among nursing home patients and is associated with cognitive decline and reduced well-being. Sleep disruptions may in part be a result of insufficient daytime light exposure. This pilot study examined the effects of dynamic “circadian” lighting and individual light exposure on sleep, cognitive performance, and well-being in a sample of 14 senior home residents. The study was conducted as a within-subject study design over five weeks of circadian lighting and five weeks of conventional lighting, in a counterbalanced order. Participants wore wrist accelerometers to track rest–activity and light profiles and completed cognitive batteries (National Institute of Health (NIH) toolbox) and questionnaires (depression, fatigue, sleep quality, lighting appraisal) in each condition. We found no significant differences in outcome variables between the two lighting conditions. Individual differences in overall (indoors and outdoors) light exposure levels varied greatly between participants but did not differ between lighting conditions, except at night (22:00–6:00), with maximum light exposure being greater in the conventional lighting condition. Pooled data from both conditions showed that participants with higher overall morning light exposure (6:00–12:00) had less fragmented and more stable rest–activity rhythms with higher relative amplitude. Rest–activity rhythm fragmentation and long sleep duration both uniquely predicted lower cognitive performance.

## 1. Introduction

Typical reported indoor lighting levels in care homes may be insufficient for proper circadian entrainment [1,2,3,4]. Light is the dominant time cue (zeitgeber) for entraining the circadian clock to local time [5,6]. A central pacemaker in the hypothalamic suprachiasmatic nucleus (SCN) generates 24 h rhythms in physiology and behavior, including sleep and activity. The SCN receives light information from intrinsically photosensitive retinal ganglion cells that contain the photopigment melanopsin [7,8,9]. In humans, the action-spectrum for non-visual light responses peaks in the blue range, between 420–480 nm [10,11,12,13,14,15].

Older adults are particularly susceptible to circadian rhythm disturbance. Aging is associated with cellular changes in the SCN, particularly in patients with dementia [16]. Additionally, age-related eye problems and retinopathies—such as senile miosis, cataracts, and optic nerve degeneration—impair ocular light transmission to the SCN [17,18,19], especially for short-wavelength blue light [20,21]. Age-related circadian rhythm disturbances include advanced circadian phase (early sleep timing), a dampening in the amplitude of circadian rhythms and more fragmented and less stable rest–activity rhythms [16,22,23,24,25,26]. Between 40 and 70% of older adults suffer from sleep disturbances, with an increased frequency in older adults with dementia [27].

Circadian rhythm disturbances and poor sleep have been linked to cognitive decline and poor health in older adults [28]. Compared to age-matched healthy adults, patients with dementia have a more fragmented and lower amplitude 24 h rest–activity rhythm [29,30,31]. Reduced amplitude, instability and fragmentation of the 24 h activity rhythm are also linked to depression in older adults [32], even when symptoms are mild, which is common in elderly persons.

Increasing zeitgeber strength may help compensate for the age-related reduced responsiveness to light. Residents of senior homes show positive effects of higher photopic illuminance on rest–activity rhythms [33,34,35,36,37,38], mood and anxiety [35,39,40]. Long-term exposure to high illuminance (e.g., 1000 photopic lux for 15–42 months) during the day has been shown to slow down cognitive deterioration in senior home residents with Alzheimer’s disease [39]. Increased light exposure has also been shown to improve sleep and cognitive performance in community-living elderly people without dementia [41]. An alternative to bright light exposure is blue-enriched lighting designed to deliver high circadian stimulation during the day. Figueiro and colleagues [35,42] found improved sleep and mood and reduced agitation following four weeks of blue enriched light (9325 K/~324 lux and 5000 K/~600 lux at the eyes) exposure in institutionalized patients with Alzheimer’s disease. Hopkins and colleagues [40] found positive (increased daytime activity and reduced anxiety) and negative (increased night-time activity, reduced sleep efficiency and quality, advanced rest–activity rhythm) effects of daytime blue-enriched lighting (17,000 K/~900 lux at 1.6 m in the direction of gaze) in senior home residents without dementia. Other lighting intervention studies in senior homes found no effects of photopic illuminance and dynamic lighting [43,44,45].

The efficacy of light is dependent on the biological time-of-day (circadian phase) of light exposure. Daytime light exposure, especially in the morning, is associated with improved sleep, mood, alertness, and cognition [36,37,39,46,47,48,49,50,51,52,53,54], whereas light exposure in the evening and night-time has been shown to have acute disruptive effects on sleep by delaying circadian timing (e.g., later sleep timing) and suppressing the release of melatonin [55,56,57]. It is thus essential to schedule light exposure in accordance with the circadian variation in the response to light. New developments in light-emitting-diode (LED) technology now enable light to be programmed (dynamic) to provide high intensity blue-enriched light during the day and low intensity, warm light at night. At present, research on dynamic lighting to promote circadian entrainment is limited. Muench and colleagues [43] found no effect of dynamic lighting on the rest–activity and melatonin profiles, emotions, and agitation behaviour in patients with severe dementia. However, they did observe a significant effect of individual light exposure, whereby residents with higher average daily light exposure showed longer emotional expression of pleasure and alertness, higher quality of life, less time spent in bed, and later bed and sleep times. Higher individual daytime light levels in senior home residents have also been shown to predict fewer night-time awakenings and a later acrophase (peak) of the daily rest–activity cycle [46].

The current pilot study examines the effect of dynamic lighting—changing in intensity (~80–1000 photopic lux) and colour temperature (~2700–5000 K) over the 24 h day to mimic the natural light/dark cycle of the sun—on sleep, cognition, and well-being (depression, fatigue, and sleep-quality) in 14 senior home residents. Participants wore wrist accelerometers to track 24 h sleep, rest–activity, and light exposure profiles over a 75 day period consisting of 5 weeks of conventional lighting (control condition, ~320 photopic lux and ~3500 K) and 5 weeks of circadian lighting (experimental condition), in a counterbalanced order. At the beginning of the study, prior to the manipulations of lighting, baseline cognitive performance was assessed. At the end of each condition, cognitive performance (week 4 of each condition) and self-reported depression, fatigue, and sleep quality (week 5 of each condition) were assessed. As the effect of lighting can take several weeks to impact physiology and behaviour, the last valid 14 days of each condition were used for statistical analyses (see Materials and Methods).

We predicted that circadian lighting, compared to conventional lighting, would (1) enhance circadian rhythm amplitude and stability of entrainment, as indicated by increased sleep duration, better sleep quality (less frequent awakenings and higher sleep efficiency), reduced rest–activity fragmentation (lower intradaily variability), stabilization of the rest–activity rhythm (higher interdaily stability), and higher relative amplitude of the rest–activity rhythm; (2) improve performance on cognitive batteries (*Pattern Comparison and Processing Speed Test*; *Flanker Inhibitory Control and Attention Test*; *Dimensional Change Card Sort Test*); and (3) improve well-being (lower depression and fatigue and better sleep quality). Given that brighter morning light conditions have been associated with improved sleep, mood, and cognition, we also predicted that participants with higher levels of morning light exposure would exhibit stronger rest–activity rhythms and better cognitive performance and well-being (lower depression and fatigue and improved sleep quality).

## 2. Results

### 2.1. Participant Demographics

We studied 14 (two male) senior home residents between the age of 70–94 years (*M* = 82.6 ± *SD* = 6.6). Participants were randomly assignment to either group A (5 weeks experimental followed by 5 weeks control) or B (5 weeks control followed by 5 weeks experimental; see Supplementary Table S1 for group demographics). The ethnic background of participants was primarily Asian (*N* = 11). Seven participants resided in assisted-living suites and seven in independent-living suites. The majority of participants were early chronotypes (see Supplementary Table S2) relative to the general adult population [58]. Participants’ chronotype, assessed using the ultra-short version of the Munich ChronoType Questionnaire (µMCTQ), ranged from extremely early (mid-sleep = 0.6 h past midnight) to intermediate (mid-sleep = 4.08 h past midnight). Our sample included participants with impaired cognitive performance. Averaged uncorrected standardized scores in *the Pattern Comparison and Processing Speed Test* were relatively low (73.07 ± 18.25, range from 47 to 103) with five participants scoring below two standard deviations from the National Institute of Health (NIH) toolbox population mean (100 ± 15).

### 2.2. Condition Effects

#### 2.2.1. Actigraphy

Actigraphy data were collected over a period of 75 days from each participant. Activity counts for each minute of the day were imported to Actiware software to generate 24 h actigraphy plots. Intervals with watch removals exceeding 5% invalid time *SW* or invalid time L (see Table 5 for definitions of study variables) were excluded, except for 24 h sleep duration where a 10% threshold was used (see Section 4). An interval refers to a time interval, which can either be manually entered into Actiware (e.g., 6:00 to 12:00) or computed by Actiware (e.g., sleep interval) based on a participants’ activity profile. After exclusions, total valid days of data collection averaged 58.4 ± 16.6 complete nights (range 24–75 nights) and 53.2 ± 16.2 complete days (range 20–72 days). 

Values for daily total and maximum white light exposure were obtained by Actiware software and averaged for each participant over the last 14 days (minimum of 10 days and nights, excluding incomplete/missing days/nights) of data collection in each condition (see Section 4). Related samples Wilcoxon signed rank tests showed that the two conditions did not differ in total or maximum white light exposure in the morning (6:00–12:00) or evening (20:00–0:00) hours (see Table 1 for sample means (*M*) and standard deviations (*SD*) and medians). During the nighttime hours (22:00–6:00), there was a significant difference (of 31 photopic lux) in maximum white light exposure between conditions, with higher white light intensities in the control condition.

Values for daily sleep variables (nocturnal sleep duration, sleep latency, wake after sleep onset (WASO), sleep efficiency) as well as 24 h sleep duration (sum of daily rest interval sleep minutes) were obtained by Actiware and averaged for each participant over the last 14 days (minimum of 10 days and nights, excluding incomplete/missing days/nights) of data per condition (see Section 4). Minute-bin activity counts were imported to Clocklab software to compute daily activity onset (AO), acrophase, as well as relative amplitude (RA), intradaily variability (IV) and interdaily stability (IS) in each condition. Incomplete days were excluded. In the case of interrupted data collection (e.g., missing days), we used the sequence with the most days (minimum of 5 consecutive days) over the last 14 days of data per condition. Definitions of dependent variables are provided in Table 5 in Section 4.

Table 2 contains sample median values of the sleep and rest–activity variables in the experimental and control conditions and Wilcoxon signed rank test z scores. Wilcoxon signed rank tests revealed no significant difference in sleep and rest–activity variables between conditions. Mixed design ANOVA comparing sleep and rest–activity variables between conditions (within-subject comparisons) with group (A vs. B based on order of condition) as a between-subject factor showed no significant effects of condition, group, or condition*group, except for acrophase, where there was a significant between-subject effect of group (*F*(1, 12) = 7.219, *p* = 0.02) and a significant interaction (*F*(1, 12) = 9.423, *p* = 0.01). Both groups delayed in acrophase by an average of 30.5 ± 36.5 min over weeks of data collection (from end August to mid-October), with group A delaying in mean acrophase from 14.01 to 14.64 (38 ± 36 min) and group B from 12.27 to 12.65 (23 ± 38 min). Levene’s test indicated homogeneous variances for both conditions.

#### 2.2.2. Cognition

NIH toolbox raw scores on the *Pattern Comparison and Processing Speed Test* and computed scores on the *Flanker Inhibitory Control and Attention Test* and *Dimensional Change Card Sort Test* were used. Two participants (one from group A and one from group B) were absent for the second assessment of cognitive batteries (travel and illness) and three participants (two from group A and one from group B) only completed the Pattern Comparison Processing Speed Test due to task difficulty. One of these three participants (group A) was able to complete the *Flanker Inhibitory Control and Attention Test* for the first (experimental condition) but not the second testing (control condition). This self-selection bias resulted in overall higher scores in the *Flanker Inhibitory Control and Attention Test and Dimensional Change Card Sort Test*.

Related samples Wilcoxon signed rank tests showed no significant difference between conditions for the three cognitive batteries (see Table 2). Mixed design ANOVA comparing scores between conditions (within subject comparison) and groups (between-subject factor) showed no effects of condition, group or group*condition for the *Pattern Comparison and Processing Speed Test* and *Flanker Inhibitory Control and Attention Test*. There was a significant between-subjects effect of group (*F*(1, 7) = 11.39, *p* = 0.012; group A had higher scores) and a trend (*p* < 0.1) for a significant group*condition interaction on the *Dimensional Change Card Sort Test* (*F*(1, 7) = 4.518, *p* = 0.071). Wilcoxon signed rank test revealed a significant improvement in computed scores in the *Dimensional Change Card Sort Test* from test session one to test session two: *z* = −2.13, *p* = 0.033, likely due to a learning effect. Additionally, we found no effect of condition on the three cognitive tests when controlling for baseline performance as a covariate in repeated design ANOVA.

#### 2.2.3. Well-Being

For each participant, we computed scores of depression (*Geriatric Depression Scale*), fatigue (*Daily Fatigue Form*), and sleep quality (*Pittsburgh Sleep Quality Index,* sleep quality item) in the control and experimental conditions. One participant was absent for the second assessment of questionnaires (illness) and one participant did not answer the sleep quality item. In the control condition, three participants reported mild depression, one moderate depression and one severe depression. It is worth noting that the two participants who reported moderate and severe depression reported only mild depression in the experimental condition. Table 2 shows sample median scores from the questionnaires in the control and experimental conditions and Wilcoxon signed rank test *z* scores. There was no significant difference in participants’ scores of depression and sleep quality. There was a trend for lower fatigue in the experimental condition, which went away after Bonferroni correction for multiple tests.

#### 2.2.4. Lighting Feedback

Table 2 shows participants’ subjective assessment of the lighting in both conditions on a 6-point scale ranging from −3 (extremely dissatisfied) to 3 (extremely satisfied). Two participants were absent for the second assessment (illness and travel). There was no significant difference in participants’ liking of the experimental versus control lighting.

### 2.3. Pooled Data: Individual Light Exposure

We computed participant averages for all sleep and activity variables over the span of their study participation (58.4 ± 16.6 nights and 53.2 ± 16.2 days). In the case of interrupted data collection (e.g., missing days), we computed separate values for each sequence of consecutive days (minimum 5 days) and averaged across sequences. We pooled uncorrected standardized scores from the three cognitive batteries and scores from the questionnaires over both conditions. See Supplementary Table S3 for sample means and standard deviations of study variables.

Participants slept an average of 6.75 ± 1.21 h a night, which is comparable to the observed sleep duration of 6.38 ± 0.95 in 1734 older adults reported by Luik and colleagues [32] (*t*(13) = 1.15, *p* = 0.270). Participants napped on average 39 ± 82 min a day (range 0–254 min). With naps included, participants slept an average of 7.41 ± 1.83 h a day. Additionally, mean sleep latency (21.42 ± 11.11, *t*(13) = 1.24, *p* = 0.235) and WASO (80.52 ± 29.24, *t*(13) = 1.42, *p* = 0.180) were comparable to the values reported by Luik and colleagues [32]. On average, participants in our sample woke up for 80.5 min a night, with a range of 47–149 min. However, compared to Luik’s sample of older adults [32], our sample had a significantly higher IV (*t*(13) = 9.387, *p* ≤ 0.000) and lower IS (*t*(13)= −7.80, *p* ≤ 0.000), indicating a more fragmented and less stable rest–activity cycle. 

Table 3 shows the Spearman’s rank correlation coefficients between participants’ total white light exposure in the morning (6:00–12:00), evening (20:00–00:00) and night (22:00–6:00) and participants’ sleep and rest–activity, processing speed (uncorrected standardized scores) and questionnaire scores (depression, fatigue, and sleep quality) pooled over both conditions. Participants with greater morning light exposure had significantly lower IV and higher IS and RA. Morning light correlations with IV, IS and RA remained significant (*p* ≤ 0.05) after controlling for activity onset with two-tailed partial correlations (*r* = −0.722 *p* = 0.005; *r* = 0.587 *p* = 0.035; *r* = 0.656 *p* = 0.015). Participants with greater morning light exposure also tended (*p* < 0.1) to have higher scores on the processing speed test. High evening light exposure correlated with shorter sleep duration and a trend (*p* < 0.1) towards later acrophase and reduced fatigue.

Table 4 contains Spearman’s rank correlation coefficients between all outcome variables. Participants with low processing speed (PS) had significantly higher IV and longer nocturnal sleep duration. Participants with processing speed scores below 70 (over 2 *SD* below the population mean) slept on average two hours (124 min) longer per night compared to residents with higher processing speeds (scores above 70).

To further explore the relationship between morning light exposure, rest–activity rhythm, and processing speed, we ran a series of forced entry multiple regressions in SPSS to predict IV, IS, RA and processing speed (see Supplementary Tables S4–S7), entering morning light (6:00–12:00) and potential confounding variables as predictors. Results show that morning light exposure predicted participants’ IV, IS and RA, after controlling for age, chronotype, depression, fatigue, and processing speed. Morning light exposure did not predict processing speed after controlling for age, IV and nocturnal sleep duration. Both IV and sleep duration uniquely predicted processing speed, independent of age and morning light exposure.

## 3. Discussion

This study examined the effects of dynamic circadian lighting—changing in intensity and colour temperature over the 24 h day—on sleep, cognitive performance, and well-being in 14 senior home residents between the age of 70–94 years. The study was conducted as a within-subject study design over 5 weeks of circadian lighting and 5 weeks of conventional lighting, in a counterbalanced order. Participants wore wrist accelerometers to track rest–activity and light exposure and completed cognitive batteries (NIH toolbox) and questionnaires (depression, fatigue, sleep quality, lighting appraisal) at the end of each condition. We found no effect of lighting condition on residents’ sleep duration, sleep latency, sleep efficiency, nocturnal awakening, and rest–activity phase, fragmentation, stability, and amplitude. Additionally, there was no effect of lighting condition on participants’ cognitive performance and well-being, and participants’ rating of both lighting conditions showed no preference for circadian over conventional lighting. Individual light exposure levels varied greatly between participants but did not differ between lighting conditions, except at night (22:00–6:00), where conventional lighting had a higher maximum light output than circadian lighting.

Our results confirm that circadian rhythms in senior home residents are highly disturbed, with greater disturbance in cognitively impaired residents [22,23,24,25,26,27,28,29,30,31]. Consistent with previous research [22,23,24,25,26], our sample had phase advanced and low amplitude rest–activity rhythms that were highly fragmented (high IV) and variable over time (low IS). We found IV to be a significant predictor of impaired cognitive performance, which is also consistent with previous research [4,29,30,31]. Fragmented activity rhythms have been observed in patients with early-onset dementia [59] and have been hypothesized to contribute to neurodegenerative processes [23,60,61,62]. Consistent with previous research [4,46], long sleep duration, independent of IV, was also a significant predictor of impaired cognitive performance, whereby participants with lower processing speed spent more time sleeping, especially at night. Circadian rhythm disturbances have been shown to be most pronounced in adults over the age of 80 years [25,63]. The majority of our sample (11/14) exceeded 80 years in age.

Pooled data from both conditions showed that participants with higher morning light exposure (6:00–12:00) had less disrupted rest–activity rhythms (less fragmented and more stable rhythms, and higher relative amplitude) even when controlling for potential confounding variables such as activity onset, age, chronotype, depression, fatigue, and cognitive performance. Although the direction of causality in this association is not known, one possible interpretation is that morning light exposure strengthens circadian entrainment and buffers cognitive decline in senior home residents. This is a cause for concern, given that older adults generally receive little daytime light exposure [1,2,3,4], particularly so for institutionalized older adults with dementia, who have been reported to experience as little as 2 min a day of bright light [3,64]. Current COVID-19 pandemic restrictions are likely to further limit older adults’ exposure to outdoor light. Of particular concern are winter months, when reported light exposure levels are even lower compared to summer months [65,66]. Decreased light exposure during winter months is also associated with a delay in circadian phase [67,68]. Consistent with this, our sample showed a significant delay in acrophase from August to October.

Our study has several limitations: First, although the number of days of data collection for each participant was high, the total participant sample size was low, which reduces the probability of detecting a treatment effect.

Second, light intensities in the treatment condition (~1000 photopic lux, horizontal plane) may have been too low to benefit circadian processes relative to the control condition. Light intensities >5000 photopic lux at the eye are recommended to treat depression, and other published lighting intervention studies have used 1000–2500 photopic lux at the eye level, in the direction of gaze, e.g., [36,39]. Zeitzer and colleagues [57] found that half of the maximal phase delaying response achieved in response to a single episode of evening bright light (~9000 photopic lux in the horizontal angle of gaze) can be obtained with just over 1% of this light (dim room light of ~100 photopic lux in the horizontal angle of gaze) with a saturating response above ∼550 photopic lux in the horizontal angle of gaze. Figueiro recommends a minimum of 400–600 photopic lux at the cornea and a correlated colour temperature (CCT) > 5000 K during the day and a maximum of 80–100 photopic lux and a CCT < 2800 K at the cornea in the evening to promote circadian entrainment [69]. It is also worth noting that the lighting levels in our control condition were ~320 photopic lux ((~30 foot-candle (fc), horizontal plane)) at 5′ below the fluorescent fixture(s) in the apartments, which is higher than typically reported lighting levels in senior homes [1]. The lighting levels in our treatment condition may provide significant benefits in cases where the control lighting levels are lower than in our study.

Another potential study limitation is that the light intervention may have been too short to elicit detectable changes in behaviour. Longitudinal studies (e.g., 1–2 years, controlled for confounding effects of aging) may give more substantive results. Nonetheless, similar studies were able to find beneficial effects of lighting within 4 weeks, e.g., [35]. Additionally, our study took place during the late summer and early autumn (August to October 2019), when people generally receive high levels of daytime light exposure, compared to winter months. Seasonal differences in light exposure have also been shown in senior home residents and affect people with dementia disproportionately [65,66]. During winter months, older adults with dementia have lower light and activity levels and greater circadian rhythm disturbance compared to age-matched healthy adults [66]. Therefore, we expect a stronger effect of artificial lighting during winter months.

A final study limitation is that our accelerometer obtained light measurements that likely underestimated true illuminance values. The Actiwatch has poor illuminance-sensing accuracy under moderately intense artificial and natural sunlight conditions. Joyce and colleagues [70] found that the Actiwatch 2 increasingly underestimated true illuminance with increasing illumination, with the highest variability between photometer and Actiwatch illuminance for illuminance values over 2500 photopic lux. In response to ~20,000 photopic lux white LED light and 30,000 photopic lux sunlight, the Actiwatch 2 underestimated illuminance by recording only 25% and 46% of the true illuminance value, respectively. While calibrated research-grade photometers provide much more accurate quantifications of light levels, they are not practical for measuring the exposure of a freely moving individual over time. Additionally, relative to a location at the plane of the cornea, photosensors worn on the wrist underestimate the actual light levels received by the eye and the circadian system [71]. Jardim and colleagues found that compared to the eye level, the measurement differences were on average 50 photopic lux higher during the day and 50 photopic lux lower at night [72]. Additionally, our wrist-worn light sensors may at times have been obstructed by clothes and/or blankets. Although we advised participants to not cover their light sensors, we did not impose any clothing restrictions.

Comparisons between our study and previous lighting intervention studies are made difficult due to different methods, including light intensity, light source, duration and timing of light exposure, and the location of the lighting (e.g., communal rooms with varying participant accessibility). Spitschan and colleagues [73] recently proposed detailed minimum guidelines on reporting light exposure in human chronobiology and sleep research. Some standardization of lighting protocols and procedures would also benefit future studies of smart circadian lighting to improve sleep, cognition, and mood in the elderly.

## 4. Materials and Methods

### 4.1. Study Site and Participants

The study took place at two adjacent buildings at a residence for seniors. The residence provides 51 one-bedroom units and 8 studios for assisted-living residents (building I) and 32 one-bedroom units for independent living (building II) residents. The majority of residents are of Japanese-Canadian descent (first and second generation) with an age range of 65–100 years. The culturally sensitive residence offers social, recreational, and spiritual activities (e.g., chair gymnastics, music, crafts, tea and chat, church service). While all residents have a large degree of independence in day-to-day living, qualified staff are available as needed for personal assistance 24 h a day. Residents supply their own furniture and are encouraged to make their suites their own home. Each apartment has a full kitchen, a living room, one bedroom, one bathroom, a balcony, and a storage space. The executive director of the residence estimated the rate of dementia to be about 15–20%.

Initially, 19 (5 male) senior residents between the age of 70–105 volunteered to participate in this study. The inclusion criteria for participants were: (1) must currently live at the study site; (2) no intention to travel during the study period; (3) able to read and write in English or Japanese to a sufficient level in order to understand the consent form and complete questionnaires; (4) competent to consent. Two participants died and three participants withdrew from the study. Their data were not included in the data analyses. The final 14 participants (2 male) were between 70–94 years (82.6 ± 6.6). The ethnic background of the participants was primarily Asian (*N* = 11). Seven participants were assisted-living (building I) and seven were independent-living residents (building II). All 14 participants lived on their own, in one-bedroom suites. Harmonized ethics committee approval by Simon Fraser University, the University of British Columbia, and Fraser Health Authority was received prior to beginning the study (REB H18-02309).

### 4.2. Study Design

The study was conducted as a within-subject study design over 75 days during summer/fall (August to October) 2019. Participants were randomly assigned to one of two groups (group A and B) to counterbalance the order of conditions. Group A received 5 weeks of circadian lighting (experimental) followed by 5 weeks of conventional lighting (control). Group B received 5 weeks of conventional lighting (control) followed by 5 weeks of circadian lighting (experimental). At the beginning of the study, demographic information and the µMCTQ were assessed in a one-on-one interview. Participants’ sleep was continuously tracked with wrist accelerometers over a span of 75 days (35 days per condition, plus a few days prior to and in-between lighting manipulations). Analyses regarding experimental effects of lighting only include actigraphy data collected during the last 14 days of each condition. Participants’ cognitive performance (three batteries from the NIH toolbox) and well-being (scales of depression, fatigue, and sleep quality) were assessed during the last week of each condition during one-on-one appointments (30–60 min) in the participants’ homes or common rooms. A baseline cognitive performance was assessed at the beginning of the study, prior to experimental light manipulation. One-on-one interviews and assistance were provided in either English or Japanese, depending on the language fluency of the participant. The study was not blinded. Participants had to approve of lighting installations inside their private living space and participants and researchers were able to recognize treatment conditions due to visible differences in emitted light in both conditions.

#### Lighting Conditions

During the 5 week experimental condition, Nano-Lit Quantum dot LED lighting (Nano-Lit Technologies, Vancouver BC, Canada) was installed in the residents’ private suite kitchen/living room area and bathroom. During the 5 week control condition, participants were exposed to their conventional lighting in the kitchen/living room area. The lighting could be switched on or off manually, but participants were asked not to use the light switch during the experimental condition (a reminder note was taped next to the light switch). To ensure uniformity between suites, new 2700 Kelvin (K) LED light bulbs were installed in all the suites’ bedroom, hallway, and dining areas (A19 60 W lamps, Philips 9.5A19/LED/827/E26/DIM 1PK 8/1) for both conditions. Additionally, a 2700 K safety smart sensor night light (Emotionlite Plugin Night Light 2700 K) was installed in the bathrooms to discourage participants from switching on the bathroom lights at night. The two buildings differed in ceiling height and number of retrofit light fixtures. Building I had a 9’ ceiling height and retrofitted 2 lighting fixtures. Building II had a ceiling height of 8′ and retrofitted a single light fixture.

**Figure 1:**
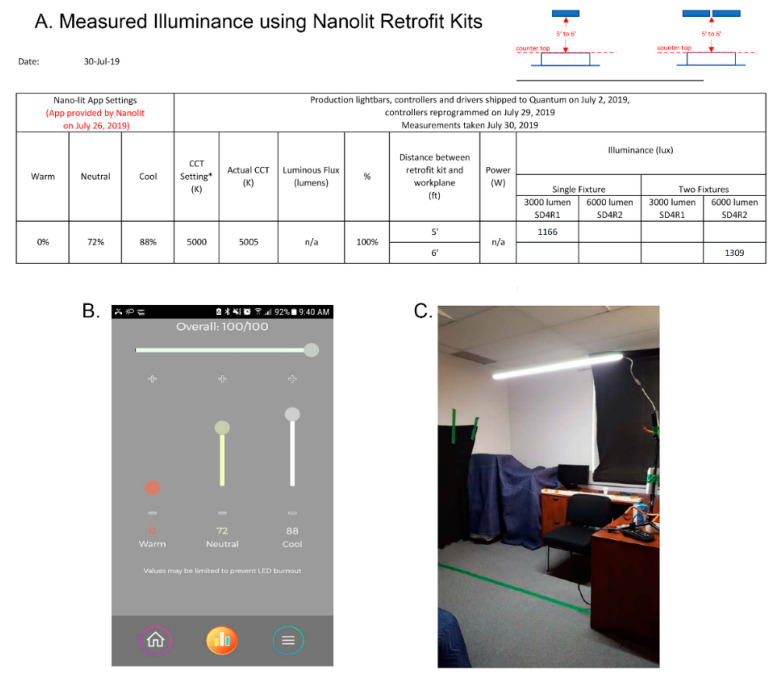
(**A**) Photometric shop light output measurements measured horizontally at 5′ and 6′ distance between retrofit plane and countertop (3′). * predicted CCT. (**B**) Screenshot of Nano-Lit app settings. (**C**) Photometric shop measurements were obtained remotely from the window so that daylight did not contribute to illuminance.

Experimental condition: in contrast to typical LED light, which uses a yellow phosphor-based coating to create white light, Quantum Dot LED lighting uses nanocrystals that can be tuned to emit all colors across the visible spectrum. This creates high-quality white light with precise color temperatures and high colour rendering. The colour rendering index (CRI) was 92 at 2700 K and at 4000 K and 97 at 6500 K. Photometric shop measurements on 30th July 2019 of the single fixture SD4R1 (3000 lumen) and double fixture SD4R2 (6000 lumen) at 100% intensity at 5000 K were completed at 6′ (building I) and 5′ (building II) from source (distance between the retrofit plane and countertop, see Figure 1). Measurements were taken remotely from the window so that daylight did not contribute to illuminance readings. Figure 1 shows the photometric shop light output measurements for double fixtures (6000 lumen) at 6′ and single fixtures (3000 lumen) at 5′. With 20% loss from existing light fixture lens, the light intensity was estimated to be 1047 photopic lux for building I and 933 photopic lux for building II. In situ measurements in one of the suites from building I, with two luminaires at 6′ distance between fixture and light meter, with the 6000 lumen Nano-Lit modules operating at 5000 K, recorded an illuminance of 915–970 photopic lux (85–90 fc). Photometric shop and in situ measurements were obtained at horizontal plane with an illuminance meter (model # 401025) by Extech Instruments.

**Figure 2:**
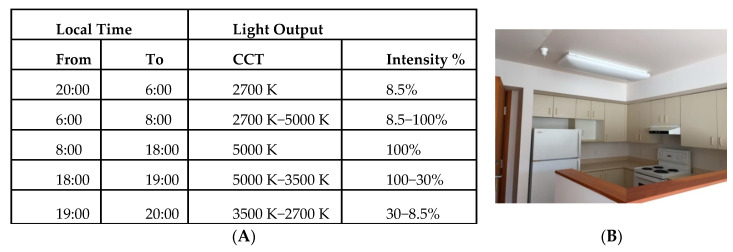
(**A**) 24 h setting (building I) of correlated colour temperature (CCT) in Kelvin (K) and % light output intensity in the experimental condition and (**B**) photograph of study site and lighting fixture.

The circadian lighting was programmed on a 24 h cycle with gradual changes in light intensity and colour temperature over the 24 h day (see Figure 2). Manufacturer obtained tabulated spectra at 10 nm spacing between 380–730 nm are reported in Table S8 in the Supplementary Materials. As recently recommended (73), the spectral irradiance was converted into absolute α-opic radiances expressed in mW/(m^2^ sr) by weighting the spectrum by the spectral sensitivity of each of the five photoreceptors separately (L, M and S cones, rods, and melanopsin-encoded intrinsically photosensitive retinal ganglion cells (ipRGCs)) and summing up the values across wavelength bands. This was obtained with CIE S 026 Toolbox v1.49a (https://doi.org/10.25039/S026.2018.TB) using the recently standardized spectral sensitivities CIE S 026/E:2018 [74]: CIE 10_cone fundamental, the CIE rod fundamental, and a standardized melanopsin spectral sensitivity. Our toolbox outputs for the 5000 K setting (8:00–18:00) are presented in the Supplementary Materials. Note, the toolbox input spectra at 5 nm spacing were extrapolated from the 10 nm spectra spacing provided to us by the manufacturer.

Control condition: during the 5 week control condition, participants were exposed to their conventional lighting in the kitchen/living room area. In situ measurements below the fluorescent fixture(s) in the apartments for both buildings measured an average of ~30 fc (320 photopic lux). Measurements were taken at night so that daylight did not contribute to illuminance readings. Measurements were made at horizontal plane with an illuminance meter (model #401025) by Extech Instruments.

### 4.3. Materials and Procedures

#### 4.3.1. Wrist Accelerometers

Daily sleep and photopic light exposure profiles were assessed with 11 Actiwatch 2 (6 in group A and 5 in group B) and 3 Spectrum devices (1 in group A and 2 in group B) by Philips Respironics, Murrysville, PA. The wrist-worn devices use accelerometers to measure movement at 32 hz, which was binned into 1 min epochs, and use a light sensor to record illumination exposures ranging from 0.01 to more than 100,000 photopic lux. Actiwatch sleep–wake algorithms have been validated with polysomnography [75,76,77,78]. The accelerometers had to be removed from the wrist while bathing and were collected by research assistants for battery charging about every 7–14 days for up to 72 h at a time.

Activity counts for each minute of the day were imported to the Philips Respironics Actiware version 6.0.9 to generate 24 h actigraphy plots to visually inspect the participants’ sleep–wake cycle and obtain daily values for sleep and light variables (see Table 5). Intervals with more than two continuous hours of missing data were excluded to prevent a time-of-day effect. We manually excluded activity data when (a) the participant was known or had reported to have removed the watch, or (b) no activity counts were registered for >2 h. Often, nocturnal sleep was interrupted by extended periods of waking. In these cases, the software chose sleep onset and wake times from the longer of two sleep bouts during the nocturnal period. If the following criteria were met, then the two sleep bouts were joined to allow reported sleep onset and wake time to represent one sleep period across the entire nocturnal period (by using the sleep onset from the earlier bout and wake time from the later bout). Criteria for combining sleep periods were (a) the period of nocturnal activity occurred during µMCTQ reported sleep times; (b) the period of nocturnal activity occurred after the lights had been switched off, determined by visual inspection of light behavior (off/on); (c) the period of nocturnal activity occurred during a time when the subject was “usually” asleep/inactive as compared to their overall actigraphy data; (d) the participant must have been asleep for at least 2 h prior to the period of awakening; (e) the duration of the nocturnal awakening had to be shorter than the shortest period of sleep.

Activity counts for each minute of the day were also imported to Clocklab version 6.1.0.1. (Actimetrics, Wilmette, IL, USA) 6 to quantify rest–activity variables using nonparametric indicators [79] by means of activity onset (AO), acrophase, intradaily variability (IV), interdaily stability (IS), and relative amplitude of the rhythm (RA). See Table 5 for variable definitions.

#### 4.3.2. Cognitive Tests

Cognitive performance was assessed with three cognitive batteries from the National Institutes of Health Toolbox Cognition Battery (NIHTB-CB) [80] presented in an app on a touch screen tablet (iPad, 6th generation) in protected cases (Avawo kids case) that can be folded back into a stand for horizontal viewing or touching position. The battery was designed to provide a set of standardized tests that could be utilized across diverse study designs and populations in large cohort studies and clinical trials from ages 3–85. The NIH Toolbox Cognition Battery (NIHTB-CB) includes subtests that evaluate processing speed, executive function, and working memory. Uncorrected standard scores compare the performance of the test takers to those in the entire NIH toolbox nationally representative normative sample (100 ± 15), regardless of age or any other variable. Although the NIHTB-CB was not designed to substitute comprehensive neuropsychological assessment, data indicate it is a valid tool for broad assessment of cognition in dementia and predementia conditions [81].

Before the study began, all participants were extensively trained on the use of touch screens and the use of cognitive batteries so as to minimize learning effects. Participants were trained and tested on the cognitive batteries during one-on-one assessments with the same research personnel in the participants’ homes or common rooms. Communication during training and testing was either in English or Japanese, depending on the language fluency of the participant. Instructions (in English) appeared visually on the monitor (and at times auditorily) and were also read aloud (and if necessary, translated) by the research personnel. All practice and test sessions started with the NIH toolbox touchscreen tutorial and each test started with a practice block during which the participants were given feedback on whether a response was correct or not. During test administration, no feedback was provided by the research personnel. Test sessions took place on Saturday and Sunday mornings, either between 10:00–11:00 or 11:00–12:00. The day and time of participants’ test sessions were kept consistent over the study. We used the following three tests from the NIHTB-CB, in the order presented below:

1. The *Pattern Comparison and Processing Speed Test* is a 3 min processing speed test. This test measures the speed of processing by asking participants to discern whether two side-by-side pictures are the same or not. The items are presented one pair at a time on the computer screen, and the participant is given 90 s to respond to as many items as possible (up to a maximum of 130 items). The participant’s raw score is the number of items answered correctly in a 90 s period, with a range of 0–130. Higher scores indicate a faster speed of processing. To evaluate simple improvement or decline over time, one can use the raw score obtained from each assessment. Slow processing speed has been associated with normal aging, with decreased processing speed being a significant contributor to age-related decline in other cognitive domains [82].
2. The *Flanker Inhibitory Control and Attention Test* is a 3 min test to assess a participant’s attention and inhibitory control. Participants are required to indicate the left–right orientation of a centrally presented arrow while inhibiting attention to the potentially incongruent stimuli that surround it. In some trials, the orientation of the flanking stimuli is congruent with the orientation of the central stimulus, and in others, it is incongruent. Performance on the incongruent trials provides a measure of inhibitory control in the context of visual selective attention. Scoring is based on a combination of accuracy and reaction time. The score provides a way of gauging raw improvement or decline from time 1 to time 2 (or subsequent assessments). This computed score ranges from 0–10, but if the score is between 0 and 5, it indicates that the participant did not score high enough in accuracy (80 percent correct or less). A change in the participant’s score from time 1 to time 2 represents real change in the level of performance for that individual since the previous assessment.
3. The *Dimensional Change Card Sort Test* is a 4 min test designed to assess cognitive flexibility/task switching. Two target pictures are presented that vary along two dimensions (e.g., shape and color). Participants are asked to match a series of bivalent test pictures (e.g., yellow balls and blue trucks) to the target pictures, first according to one dimension (e.g., color) and then, after a number of trials, according to the other dimension (e.g., shape). “Switch” trials are also employed, in which the participant must change the dimension being matched. It consists of four blocks (practice, pre-switch, post-switch, and mixed). Scoring is based on a combination of accuracy and reaction time. The computed score provides a way of gauging raw improvement or decline from time 1 to time 2. This computed score ranges from 0–10, but if the score is between 0 and 5, it indicates that the participant did not score high enough in accuracy (80 percent correct or less). A change in the participant’s score from time 1 to time 2 represents real change in the level of performance for that individual since the previous assessment.

#### 4.3.3. Standardized Questionnaires

At the beginning of the study, we collected demographic information (participant age, gender, ethnicity, and whether they were living alone or not) and administered a chronotype questionnaire during one-on-one interviews with the participants in common rooms. We assessed participants’ well-being (depression, fatigue, and sleep quality) and lighting appraisal with questionnaires (see below) during the final week of each condition. Questionnaires were provided in English and/or Japanese language on iPads (6th generation) during one-on-one appointments with the same research personnel, in the participants’ homes or in common rooms. The participants were asked to complete the questionnaires on their own but were provided assistance from research personnel on a need basis. Assistance was provided in English and/or Japanese.

Sleep timing and chronotype were assessed with the ultra-short version of the *Munich ChronoType Questionnaire* (µMCTQ) [83]. The µMCTQ contains simple questions on the current timing of sleep and wake behaviour. As our participants have no workdays, we did not ask participants to report sleep patterns separately for work and free days. Instead, we asked them to report usual sleep patterns. Chronotype was assessed by means of mid-sleep.

Depression was assessed with the shortened form of the *Geriatric Depression Scale* [84], a 15-item self-report yes/no inventory in which participants rate the extent to which they have been bothered by various symptoms. Of the 15 items, 10 indicate the presence of depression when answered positively, while the rest indicate depression when answered negatively. Scores of 0–4 are considered normal, depending on age, education, and complaints; 5–8 indicate mild depression; 9–11 indicate moderate depression; and 12–15 indicate severe depression. The short form is more easily used by physically ill and mildly to moderately demented patients who have short attention spans and/or feel easily fatigued. It takes about 5 to 7 min to complete.

Fatigue was assessed with the short version of the Daily Fatigue Form [85]. The 7-item scale describes daily fatigue in the last day along a 5-point scale ranging from 1 (*never*) to 5 (*always*). Sample item: “How often did you feel tired?”

Sleep quality was assessed with one item from the Pittsburgh Sleep Quality Index [86]: “In the past month how would you rate your sleep quality?” on a 4-point scale from very good (0) to very bad (3).

Lighting appraisal was assessed with a seven-point Likert scale (0 = very unsatisfied, 3 = neutral, 6 = very satisfied): “How satisfied are you with the lighting?”, which was asked separately for the bedroom, living room, kitchen, and bathroom [48].

### 4.4. Statistical Analyses

All statistical analyses were computed in SPSS version 25. Due to the small sample size (*N* = 14), we primarily used non-parametric statistical analyses as they are based on fewer assumptions (e.g., normal distribution). Correlations between variables were analyzed with Spearman’s rho and when comparing between experimental conditions, we performed related samples Wilcoxon signed rank tests. We used parametric tests to determine unique predictors of outcome variables by means of multiple regression and to compare conditions (within subject comparisons) while controlling for potential confounding variables (e.g., order of condition) by means of mixed design ANOVA. The significance level was set at *α* = 0.05. Due to a priori predictions of effect direction, we also report as statistical trends those cases in which *p* > 0.05 but < 0.1. Assuming an effect size (*d*) of 0.5 and power of 0.8, with α = 0.05, a priori power analyses with G*Power [87] estimated a required sample size of *n* = 35 for two-tailed predictions and *n* = 28 for one-tailed predictions.

## Figures and Tables

**Table 1:** Light exposure per condition and Wilcoxon signed rank test comparisons.

Total White Light Exposure (Lux-Minutes)					
Time of Day	Control Condition		Experimental Condition		Wilcoxon Signed Rank Test
	M ± SD	Median	M ± SD	Median	
6:00–12:00	37,113 ± 38,129	27,947	65,444 ± 114,04	14,247	*z* = −0.22, *p* = 0.826
20:00–0:00	3127 ± 4385	978	1995 ± 2242	711	*z* = −1.41, *p* = 0.158
22:00–6:00	1116 ± 2.551	202	789 ± 1328	231	*z* = −0.94, *p* = 0.345
**Maximum White Light Exposure (lux)**					
**Time of day**	**Control Condition**		**Experimental Condition**		**Wilcoxon Signed Rank Test**
	**M ± SD**	**Median**	**M ± SD**	**Median**	
6:00–12:00	4216 ± 4607	3383.65	5240 ± 8009	1717	*z* = −0.22, *p* = 0.826
20:00–0:00	188 ± 222	90.45	176 ± 203	82	z = −1.54, p = 0.124
22:00–6:00	79 ± 118	35.53	48 ± 82	19	z = −2.48, p = 0.013

Light exposure levels were averaged over the last valid 14 days per condition (*n* = 14), *α* = 0.05.

**Table 2:** Sample median values of study variables per condition and Wilcoxon signed rank tests.

Sleep and Rest-Activity (*n* = 14)	Control Condition	Experimental Condition	Wilcoxon Signed Rank Test
24-h Sleep Duration (Minutes)	444.68	430.24	*z* = −0.23, *p* = 0.814
Sleep Duration (Minutes)	406.50	407.24	*z* = −0.53, *p* = 0.594
Sleep Latency (Minutes)	20.88	15.66	*z* = −0.34, *p* = 0.730
Wake After Sleep Onset (Minutes)	77.11	81.11	*z* = −0.28, *p* = 0.778
Sleep Efficiency	77.78	76.41	*z* = −0.09, *p* = 0.925
Acrophase	13.13	13.23	*z* = −0.53, *p* = 0.594
Relative Amplitude (RA)	0.74	0.76	*z* = -1.02, *p* = 0.308
Intradaily Variability (IV)	1.00	1.10	*z* = −0.59, *p* = 0.552
Interdaily Stability (IS)	0.57	0.51	*z* = −0.35, *p* = 0.972
**Cognitive Batteries**			
Pattern Comparison and Processing Speed Test (Raw score; *n* = 12)	32	30	*z* = −0.08, *p* = 0.937
Flanker Inhibitory Control and Attention Test (Computed Score; *n* = 9)	7.14	7.14	*z* = −1.12, *p* = 0.260
Dimensional Change Card Sort Test (Computed Score; *n* = 9)	6.27	6.39	*z* = −0.41, *p* = 0.678
**Geriatric Depression Total Score (*n* = 13)**			
None (0–4)	9	8	
Mild (5–8)	3	5	
Moderate (9–11)	1	0	
Severe (12–15)	1	0	
**Well-Being**			
Geriatric Depression Scale (*n* = 13)	3.00	3.00	*z* = −0.36, *p* = 0.720
Daily Fatigue Form (*n* = 13)	3.38	3.28	*z* = −1.68, *p* = 0.092
Sleep Quality (*n* = 12)	2.00	1.00	*z* = −0.54, *p* = 0.589
**Lighting Feedback (*n* = 13)**			
Bedroom	2.00	2.00	*z* = −0.21, *p* = 0.833
Kitchen	2.00	2.00	*z* = −0.21, *p* = 0.833
Living Room	2.00	2.00	*z* = −1.03, *p* = 0.302
Bathroom	2.00	2.00	*z* = −0.54, *p* = 0.590

Participants’ sleep and rest–activity variables were averaged over the last valid 14 days of study participation in each condition (minimum 10 days). Geriatric depression total scores range from 0 to 15, with scores 5–8 = mild depression; 9–11 = moderate depression; and 12–15 = severe depression. Fatigue scores ranged from 0 (no fatigue) to 5 (high fatigue). Sleep quality scores range from 0 (very good) to 3 (very bad). Lighting responses were scored on a 6-point scale ranging from −3 (extremely dissatisfied) to 3 (extremely satisfied). *α* = 0.05.

**Table 3:** Correlations between white light exposure and main outcome variables.

Outcome Variables	Total White Light Exposure		
	6:00–12:00	20:00–0:00	22:00–6:00
IV	−0.749 **	−0.037	−0.257
IS	0.551 *	−0.183	0.176
RA	0.666 **	−0.029	0.187
Acrophase	0.112	0.499 °	0.297
Nocturnal Sleep Duration	−0.279	−0.657 **	−0.371
Processing Speed	0.503 °	0.415	0.147
Depression	0.039	−0.047	0.210
Fatigue	−0.075	−0.462 °	−0.002
Sleep Quality	0.238	0.242	0.419

Spearman’s ρ correlation coefficients (*n* = 14) between total white light exposure and intradaily variability (IV), interdaily stability (IS), relative amplitude (RA), acrophase, nocturnal sleep duration, processing speed, depression, fatigue and sleep quality. High values of sleep quality indicate low sleep quality. ° *p* ≤ 0.1; * *p* ≤ 0.05; ** *p* ≤ 0.01.

**Table 4:** Correlations between main study variables.

	IV	IS	RA	Acroph.	SD	PS	Depr.	Fatigue	SQ
**IV**		−0.579 *	−0.626 *	−0.095	0.332	−0.534 *	−0.097	−0.055	−0.367
**IS**	−0.579 *		0.478 °	−0.405	0.240	0.101	0.046	0.085	0.309
**RA**	−0.626 *	0.478 °		−0.319	0.042	0.187	0.108	0.097	0.235
**Acroph.**	−0.095	−0.405	−0.319		−0.582 *	0.209	0.379	0.102	0.353
**SD**	0.332	0.240	0.042	−0.582 *		−0.670 **	0.111	0.130	−0.041
**PS**	−0.534 *	0.101	0.187	0.209	−0.670 **		0.202	−0.133	0.244
**Depr.**	−0.097	0.046	−0.108	0.379	0.111	−0.202		0.546 *	0.323
**Fatigue**	−0.055	0.085	0.097	−0.102	0.130	−0.133	0.546 *		0.195
**SQ**	−0.367	0.309	0.235	0.353	−0.041	0.244	0.323	0.195	

Spearman’s ρ correlation coefficients (*N* = 14) between intradaily variability (IV), interdaily stability (IS), relative amplitude (RA), acrophase (Acroph.), nocturnal sleep duration (SD), processing speed (PS), depression (Depr.), fatigue, and sleep quality (SQ). High values of sleep quality indicate low sleep quality. ° *p* ≤ 0.1; * *p* ≤ 0.05; ** *p* ≤ 0.01.

**Table 5:** Definitions of sleep, light, and rest–activity variables.

Sleep Variables (Actiware)	Definition
Nocturnal Rest Interval Duration	The time elapsed between the start time and the end time of Actiware (or manually, see Data Cleaning) scored Nocturnal Rest Interval, in minutes.
Sleep Latency (SL)	The time required for sleep to start after initiating the intent to sleep. The time between the start of Nocturnal Rest Interval and the Nocturnal Sleep Interval start time, in minutes, and is controlled by the sleep interval detection algorithm
Wake After Sleep Onset (WASO)	The total number of epochs between the start time and the end time of the Nocturnal Sleep Interval scored as wake by Actiware (or manually, see data cleaning) multiplied by the epoch length in minutes.
Sleep Efficiency	The percentage of time spent in bed sleeping. Sleep Duration divided by Nocturnal Rest Interval Duration minus total invalid time (sleep/wake) of the Nocturnal Rest Interval multiplied by 100.
Nocturnal Sleep Duration	The total number of epochs for the nocturnal interval scored as sleep by Actiware (or manually, see data cleaning) multiplied by the epoch length in minutes.
24-h Sleep Duration	Added minutes of sleep during Actiware scored nocturnal and daytime Rest Intervals from 12:00–12:00
Invalid Time SW	The total number of epochs for the given interval for which the sleep/wake scoring algorithm did not have enough data to determine a sleep or wake score multiplied by the epoch length in minutes.
**Light Variables (Actiware)**	
Total White Light Exposure	The sum of all white light values for the given interval multiplied by the epoch length in minutes, expressed in lux-minutes.
Maximum White Light Exposure	The largest white light intensity value for the given interval expressed in lux.
Invalid Time L	The total number of epochs for the given interval for which the light value is invalid. This may occur under multiple circumstances including excluded intervals, device error, communication error, data corruption, time the logger is in the docking station, or time between data collection sessions.
**Rest–Activity Variables (Clocklab)**	
Activity Onset (AO)	The time of day corresponding to the onset of activity in a 24 h time series.
Acrophase (AP)	The time of day corresponding to the peak of a sine wave fitted to 24 h time series of activity.
Relative Amplitude (RA)	The ratio of the most active 10 h period to the least active 5 h period across the averaged 24 h profile, starting at 0:00. Values range from 0 to 1 with higher values indicating a higher amplitude sleep–wake cycle, with a consolidated period of low activity during sleep (L5) and a consolidated period of high activity during the day (M10), which is considered healthy in individuals. Relative amplitude reflects the difference between M10 activity and L5 activity and is calculated as follows: (M10 − L5)/(M10 + L5).
Intradaily Variability (IV)	Fragmentation of the rhythm relative to its 24 h amplitude (score). It is a measure of circadian rhythm disturbance, as it quantifies the fragmentation of periods of activity from periods of rest within a 24 h period. IV scores range from 0 to 2 and are typically below 1. High Intradaily Variability may indicate daytime napping and/or nighttime arousals.
Interdaily Stability (IS)	Is a measure of the strength of circadian rhythmicity, the degree of consistency of activity patterns from one day to the next. IS was computed per condition. IS represents the degree of consistency of activity patterns from 1 day to the next. Interdaily stability is the 24 h value from a chi square periodogram, normalized for the number of data points. Interdaily stability scores range from 0 to 1, and may typically be 0.6, with lower scores representing poor consistency of activity patterns. Quantifies the synchronization to the 24 h light-dark cycle.

## Supplementary Materials

The following are available online at https://www.mdpi.com/2624-5175/2/4/40/s1, Table S1: Demographic statistics following random assignment into groups A and B; Table S2: Frequency of chronotype categories based on MCTQ assessed mid-sleep at the start of the study; Table S3: Sample mean and standard deviation of study variables; Table S4: Multiple regression predicting IV; Table S5: Multiple regression predicting IS; Table S6: Multiple regression predicting RA; Table S7: Multiple regression predicting processing speed; Table S8: Manufacturer tabulated spectra; Figure S1: Tabulated spectra at 2812 K (20:00–6:00), 3847 K (7:00), 5050 K (8:00–18:00) and 3446 K (19:00). Additional observation—concern for nighttime wandering; and the CIE S 026 α-opic toolbox outputs.

## Abbreviations

SCN	suprachiasmatic nucleus
µMCTQ	ultra-short version of the Munich ChronoType Questionnaire
NIHTB-CB	National Institutes of Health Toolbox-Cognition Battery
RA	relative amplitude
IV	intradaily variability
IS	interdaily stability
CCT	correlated colour temperature
CRI	colour rendering index
