# Supplementary material for: Light in the Senior Home: Effects of Dynamic and Individual Light Exposure on Sleep, Cognition, and Well-Being
Source: Clocks Sleep. 2020 Dec 14;2(4):557–76. doi: 10.3390/clockssleep2040040 (PMC7768397; doi:10.3390/clockssleep2040040)
Supplement: Supplementary file 1 [file clockssleep-02-00040-s001.pdf]

Table S1. Demographic statistics following random assignment into groups A and B.

**Group**	**Sample Size (*N*)**	**Female (*N*)**	**Age in years (Mean +/- *SD*)**	**Assisted-Living (*N*)**	**MCTQ Midsleep (*Mean* +/- *SD*)**	**Pattern Comparison and Processing Speed Test, uncorrected standardized scores (*Mean* +/- *SD*)**
A	7	6	81.29 +/- 5.12	4	3.31 +/- 0.86	71.71 +/- 19.98
B	7	6	83.86 +/- 8.07	3	2.15 +/- 1.00	74.43 +/- 17.83

Table S2. Frequency of chronotype categories based on MCTQ assessed mid-sleep at study begin.

**Chronotype**	***N***
Extremely Early (MS < 1.49)	2
Moderately Early (MS = 1.5 to 2.49)	2
Slightly Early (MS= 2.5 to 3.49)	7
Intermediate (MS= 3.5 to 4.49)	3
Slightly Late (MS= 4.5 to 5.49)	0
Moderately Late (MS =5.5 to 6.49)	0
Extremely Late (MS > 6.5)	0

Chronotype categories from the Ultra Short Munich ChronoType Questionnaire (μMCTQ) assessed mid-sleep (MS) at study begin

Table S3. Sample mean and standard deviation of study variables

**Study Variables**	***M* +/- *SD***
24-Hour Sleep Duration (Minutes)	444.43 +/- 109.91
Nocturnal Sleep Duration (Minutes)	405.23 +/- 72.84
Sleep Latency (Minutes)	21.42 +/- 11.11
Wake After Sleep Onset (Minutes)	80.52 +/- 29.24
Sleep Efficiency	77.63 +/ 4.64
Acrophase	13.35 +/- 1.58
Relative Amplitude (RA)	0.76 +/- 0.09
Intradaily Variability (IV)	1.07 +/- 0.26
Interdaily Stability (IS)	0.57 +/- 0.11
Pattern Comparison and Processing Speed Test (*N*=14)	73.07 +/- 18.25
Flanker Inhibitory Control and Attention Test (*N*=12)	82.08 +/- 19.25
Dimensional Change Card Sort Test (*N*=11)	90.50 +/- 6.62
Geriatric Depression Scale (*N*=13)	4.08 +/- 2.74
Daily Fatigue Form (*N*=13)	3.00 +/- 0.80
Sleep Quality (*N*=12)	1.25 +/- 0.70

Means (*M*) and standard deviations (*SD*) from actigraphy variables were averaged over days of study participation (58.4 ± 16.6 nights and 53.2 ± 16.2 days). Cognitive batteries and questionnaires were pooled over both conditions. Cognitive test results are reported in uncorrected standardized scores. Depression scores range from 0 (low) to 15 (high), with scores 5-8 = mild depression; 9-11 = moderate depression; and 12-15 = severe depression). Fatigue scores range from 0 (no fatigue) to 5 (high fatigue). Sleep quality scores range from 0 (very good) to 3 (very bad)

Table S4. Multiple Regression predicting IV

	Standardized coefficient b	*t*	*Sig.*
**Constant**		1.735	.133
**Morning Light**	-.734	-3.069	.022
**Age**	-.002	-0.006	.995
**Chronotype**	.062	0.210	.840
**Depression**	.031	0.083	.936
**Fatigue**	-.280	-0.863	.421
**Processing Speed**	-.451	-1.982	.095

Multiple Regression with intradaily variability (IV) as outcome and morning light, age, chronotype, depression, fatigue and processing speed as predictors. n=14, α=0.05.

Table S5. Multiple Regression predicting IS

	Standardized coefficient b	*t*	**Sig.**
**Constant**		0.206	.844
**Morning Light**	.757	2.579	.042
**Age**	.209	0.644	.544
**Chronotype**	-.498	-1.388	.215
**Depression**	.121	0.268	.798
**Fatigue**	.241	0.605	.567
**Processing Speed**	.188	0.673	.526

Multiple Regression with interdaily stability (IS) as outcome and morning light, age, chronotype, depression, fatigue and processing speed as predictors. n=14, α=0.05.

Table S6. Multiple Regression predicting RA

	Standardized coefficient b	*t*	*Sig.*
**Constant**		0.943	.382
**Morning Light**	.751	2.473	.048
**Age**	.080	0.238	.820
**Chronotype**	-.128	-0.344	.743
**Depression**	-.315	-0.675	.525
**Fatigue**	.415	1.006	.353
**Processing Speed**	.218	0.754	.480

Multiple Regression with relative amplitude (RA) as outcome and morning light, age, chronotype, depression, fatigue and processing speed as predictors. n=14, α=0.05.

Table S7. Multiple Regression predicting Processing Speed

	Standardized coefficient b	*t*	*Sig.*
**Constant**		2.892	.011
**Age**	.054	0.269	.794
**Morning Light**	-.299	-1.097	.301
**IV**	-.610	-2.371	.042
**Sleep Duration**	-.635	-3.365	.008

Multiple Regression with processing speed as outcome and age, morning light, intradaily variability (IV) and sleep duration as predictors. n=14, α=0.05.

Table S8. Manufacturer Tabulated Spectra

uW/cm2/nm at 4 foot distance horizontal plane				
**Wavelength**	**Setting**			
	**20:00-6:00 2812K**	**7:00 3847K**	**8:00-18:00 5050K**	**19:00 3446K**
**380**	0.05	0.04	0.02	0.05
**390**	0.05	0.04	0.02	0.05
**400**	0.05	0.03	0.02	0.05
**410**	0.16	0.10	0.04	0.17
**420**	0.25	0.21	0.14	0.28
**430**	0.24	0.41	0.52	0.36
**440**	0.28	1.05	1.58	0.65
**450**	0.33	1.53	2.23	0.88
**460**	0.31	1.15	1.61	0.71
**470**	0.27	0.78	1.09	0.53
**480**	0.25	0.71	1.04	0.49
**490**	0.26	0.84	1.32	0.54
**500**	0.27	1.02	1.69	0.63
**510**	0.28	1.17	2.01	0.70
**520**	0.27	1.28	2.26	0.74
**530**	0.26	1.35	2.46	0.76
**540**	0.25	1.40	2.60	0.77
**550**	0.24	1.42	2.73	0.77
**560**	0.24	1.43	2.83	0.77
**570**	0.23	1.45	2.96	0.77
**580**	0.23	1.47	3.13	0.78
**590**	0.22	1.50	3.34	0.79
**600**	0.22	1.55	3.58	0.80
**610**	0.22	1.60	3.83	0.83
**620**	0.21	1.62	3.98	0.83
**630**	0.20	1.52	3.85	0.78
**640**	0.19	1.31	3.42	0.69
**650**	0.18	1.08	2.90	0.58
**660**	0.16	0.87	2.39	0.48
**670**	0.14	0.69	1.93	0.39
**680**	0.12	0.54	1.53	0.31
**690**	0.10	0.42	1.20	0.25
**700**	0.08	0.32	0.93	0.19
**710**	0.06	0.25	0.71	0.15
**720**	0.05	0.19	0.54	0.12
**730**	0.04	0.14	0.41	0.09

Spectra in tabulated form at 10 nm spacing between 380 and 730 nm for 2812K (20:00-6:00), 3874K (7:00), 5050K (8:00-18:00) and 3446K (19:00). Assessed with the hardware i1 studio at 4 foot distance from source.

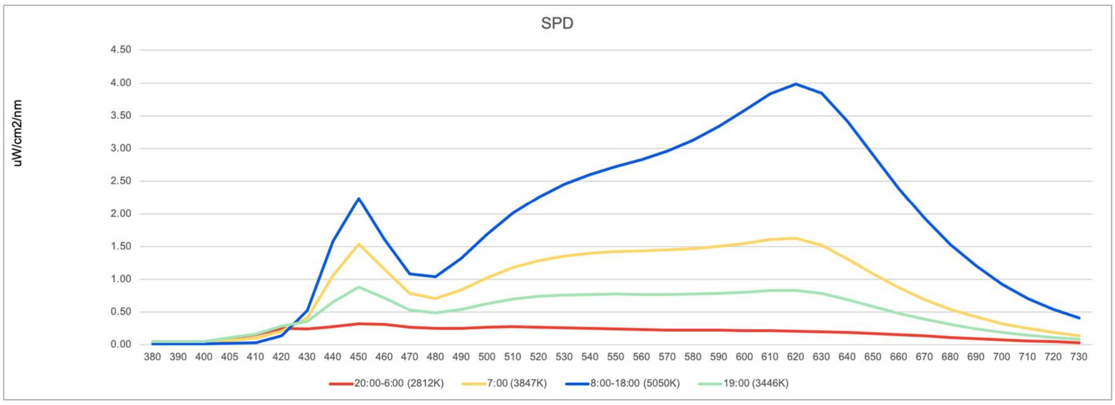

Figure S1. Tabulated Spectra at 2812K (20:00-6:00), 3847K (7:00), 5050K (8:00-18:00) and 3446K (19:00).

Additional Observation – Concern for nighttime wandering.

We would like to highlight a concern about the potential negative effects of nighttime lighting in residential care homes. During the study, it was brought to our attention that one of the participants was experiencing higher than usual nighttime wandering in the circadian lighting (experimental) condition. The nurse attributed this to the increased light levels in the kitchen/living room area at night. Under usual circumstances, outside of the study, the nurses switched the lights off in this unit every night, except for a dim closet light. In the experimental condition, nighttime light levels in the kitchen/living room area were tuned to a recommended ~80 photopic lux at 2700K to minimize visibility concerns at night. We had asked participants to leave lights on 24/h a day (reminder notes were taped above the light switches) so as to keep extraneous light manipulations to a minimum and avoid participants forgetting to switch the lights on in the morning. As residents were sleeping in their bedroom, where light switches could be used freely, we did not think this would be a concern. However, in this particular case, the change in nighttime lighting in the kitchen/living room area (which typically was switched off at night and back on in the morning by a nurse) caused the resident to erroneously assume it was daytime, which led her to start her day in the middle of her biological night (e.g. get dressed and walk to the breakfast area). We therefore recommend future studies to carefully assess usual lighting behavior in the residents and to consult carefully with nurses.

CIE S 026 α-Opic Toolbox Outputs

CIE S 026 α-opic Toolbox - v1.049a - 2020/11

Outputs Please note the α-opic Toolbox is not part of CIE S 026. See Disclaimer sheet.

Values for: Nano-Lit (5050K) — INPUTS Standard CIE S 026 calculations.

Scaling factor for inputs: 1E-02

**irradiance W.m-2**	**illuminance lx**	**photon irradiance log Q/(s-1.m-2)**

*(unweighted) irradiance = ∫ spectral irradiance * dλ*

*(photopic) illuminance = K_m * ∫ spectral irradiance * V(λ) * dλ* , where $K_m \approx 683$ *lm.W*$^{-1}$

*(unweighted) photon irradiance = ∫ spectral photon irradiance * dλ*

irradiance, W.m-2	illuminance, lx	log photon irradiance/(s-1.m-2)
6.67	2046.53	19.290

α-opic irradiance, W.m-2 — **α-opic irradiance**

*α-opic irradiance = ∫ spectral irradiance * α-opic action spectrum * dλ*

S-cone-opic	*M-cone-opic*	*L-cone-opic*	*Rhodopic*	***Melanopic***
0.71	2.50	3.36	1.85	**1.48**

α-opic efficacy of luminous radiation, mW.lm-1 — **α-opic ELR**

α-opic ELR = α-opic irradiance / illuminance

S-cone-opic	*M-cone-opic*	*L-cone-opic*	*Rhodopic*	***Melanopic***
0.3453	1.2218	1.6417	0.9019	**0.7235**

α-opic equivalent daylight (D65) illuminance, lx — **α-opic EDI**

α-opic EDI = α-opic irradiance / α-opic ELR for daylight (D65)

S-cone-opic	*M-cone-opic*	*L-cone-opic*	*Rhodopic*	***Melanopic***
864.74	1717.49	2062.56	1273.21	**1116.46**

log α-opic photon irradiance, log Q/(s-1.m-2), where — **α-opic photon irradiance**

*α-opic photon irradiance = ∫ spectral photon irradiance * photon system α-opic action spectrum * dλ*

S-cone-opic	*M-cone-opic*	*L-cone-opic*	*Rhodopic*	***Melanopic***
18.202	18.833	18.982	18.672	**18.562**

α-opic photon irradiance in standard notation

e.g. 3.646E+18 = 10^18.562, with rounding to 3 decimal places

photon irradiance, s-1.m-2: 1.948E+19

α-opic photon irradiance, s-1.m-2

1.592E+18	6.806E+18	9.588E+18	4.699E+18	**3.646E+18**

CIE S 026 α-opic Toolbox - v1.049a - 2020/11

Charts Please note the α-opic Toolbox is not part of CIE S 026. See Disclaimer sheet.

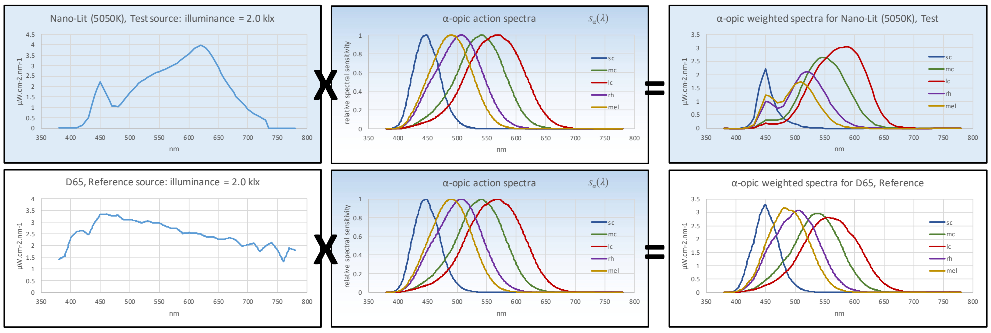

The input data is called the Test source. The default CIE S 026 Reference source is daylight (D65); Lucas et al., 2014 uses equi-energy (E).
The units used on the y-axis depend on the prefixes selected in Inputs cells C12 and C13. The units used in the chart titles depend on the prefixes selected in Advanced Outputs cells H10 and H11.

CIE S 026 α-opic Toolbox - v1.049a - 2020/11

Select current "CIE S 026" or old "Lucas et al., 2014" quantities: CIE S 026

Advanced Outputs Please note the α-opic Toolbox is not part of CIE S 026. See Disclaimer sheet.

Inputs = blue

	Inputs			Outputs	
Basis of system:	**radiant flux (power), W**	Basis of system:	**radiant flux (power), W**	**luminous flux, lm**	**photon flux, s-1**
Quantity, Q:	**irradiance**	Quantity, Q:	**irradiance**	**illuminance**	**photon irradiance**
Units:	**µW.cm-2**	Units:	**µW.cm-2**	**klx**	**log Q/(s-1.cm-2)**
Output prefix	µ	Select:	µ	Select: k	
Area prefix	c	Select:	c	Do not use:	Select: c
Scaling factors	1E-02		1E+00	1E-05	1E-06

INPUTS: Standard CIE S 026 calculations.

Note to (T2) $K_m \approx K_{cd} = 683$ lm.W^{-1}

*(unweighted) irradiance = ∫ spectral irradiance * dλ* (T1)

*(photopic) illuminance = K_m * ∫ spectral irradiance * V(λ) * dλ* (T2)

*(unweighted) photon irradiance = ∫ spectral photon irradiance * dλ* (T3)

Values for		irradiance, µW.cm-2	illuminance, klx	log photon irradiance/(s-1.cm-2)
Nano-Lit (5050K)	Test source	667.19	2.05	15.290
daylight (D65)	Reference: "D"	999.17	2.05	15.454

α-opic irradiance, µW.cm-2 **α-opic irradiance**

*α-opic irradiance = ∫ spectral irradiance * α-opic action spectrum * dλ* (T4)

α-opic irradiance for		*S-cone-opic*	*M-cone-opic*	*L-cone-opic*	*Rhodopic*	***Melanopic***
Nano-Lit (5050K)		70.67	250.04	335.97	184.58	**148.07**
daylight (D65)		167.26	297.94	333.36	296.69	**271.41**

α-opic efficacy of luminous radiation, mW.lm-1 **α-opic ELR**

α-opic ELR = α-opic irradiance / illuminance (T5)

α-opic ELR for		*S-cone-opic*	*M-cone-opic*	*L-cone-opic*	*Rhodopic*	***Melanopic***
Nano-Lit (5050K)		0.3453	1.2218	1.6417	0.9019	**0.7235**
daylight (D65)		0.8173	1.4558	1.6289	1.4497	**1.3262**

α-opic daylight (D65) efficacy ratio, 1 **α-opic DER**

α-opic DER = α-opic ELR / α-opic ELR for daylight (D65) (T6)

α-opic DER for		*S-cone-opic*	*M-cone-opic*	*L-cone-opic*	*Rhodopic*	***Melanopic***
Nano-Lit (5050K)		0.4225	0.8392	1.0078	0.6221	**0.5455**
daylight (D65)		1.0000	1.0000	1.0000	1.0000	**1.0000**

α-opic equivalent daylight (D65) illuminance, klx **α-opic EDI**

α-opic EDI = α-opic irradiance / α-opic ELR for daylight (D65) (T7)

*α-opic EDI = illuminance * α-opic DER* (T8)

α-opic EDI for		*S-cone-opic*	*M-cone-opic*	*L-cone-opic*	*Rhodopic*	***Melanopic***
Nano-Lit (5050K)		0.86	1.72	2.06	1.27	**1.12**
daylight (D65)		2.05	2.05	2.05	2.05	**2.05**

log α-opic photon irradiance, log Q/(s-1.cm-2), where **α-opic photon irradiance**

*α-opic photon irradiance = ∫ spectral photon irradiance * photon system α-opic action spectrum * dλ* (T9)

α-opic photon irradiance for		*S-cone-opic*	*M-cone-opic*	*L-cone-opic*	*Rhodopic*	***Melanopic***
Nano-Lit (5050K)		14.202	14.833	14.982	14.672	**14.562**
daylight (D65)		14.576	14.909	14.978	14.878	**14.825**

CIE S 026 α-opic Toolbox - v1.049a - 2020/11

Glossary Please note the α-opic Toolbox is not part of CIE S 026. See Disclaimer sheet.

List of quantities, abbreviations and symbols

Previously published (CIE DIS 017:2016; CIE 018:2019)

E_v = (photopic) illuminance; L_v = (photopic) luminance
$E = E_e$ = irradiance (*i.e.* unweighted); $L = L_e$ = radiance (*i.e.* unweighted)
E_p = photon irradiance (*i.e.* unweighted); L_p = photon radiance (*i.e.* unweighted)

From CIE S 026:2018

α-opic (α) may represent any one of S-cone-opic (sc), M-cone-opic (mc), L-cone-opic (lc), rhodopic (rh) and melanopic (mel)
$s_\alpha(\lambda) = s_{e,\alpha}(\lambda)$ = α-opic spectral weighting function (action spectrum)

$K_{\alpha,v}$ = α-opic efficacy of luminous radiation, **α-opic ELR**
$K^{D65}_{\alpha,v}$ = α-opic ELR for daylight (D65)

$\gamma^{D65}_{\alpha,v}$ = α-opic daylight (D65) efficacy ratio, **α-opic DER**

$E_\alpha = E_{e,\alpha}$ = α-opic irradiance (*i.e.* weighted by $s_\alpha(\lambda)$)
$E^{D65}_{v,\alpha}$ = α-opic equivalent daylight (D65) illuminance, **α-opic EDI**

$L_\alpha = L_{e,\alpha}$ = α-opic radiance (*i.e.* weighted by $s_\alpha(\lambda)$)
$L^{D65}_{v,\alpha}$ = α-opic equivalent daylight (D65) luminance, **α-opic EDL**

From CIE S 026:2018 and 9th edition of SI Brochure

$s_{p,\alpha}(\lambda)$ = α-opic spectral weighting function (action spectrum) in the photon system (renormalised to maximum of 1)
$E_{p,\alpha}$ = α-opic photon irradiance (*i.e.* weighted by $s_{p,\alpha}(\lambda)$)
$L_{p,\alpha}$ = α-opic photon radiance (*i.e.* weighted by $s_{p,\alpha}(\lambda)$)

Further α-opic quantities and their symbols can be derived, *e.g.* α-opic equivalent daylight (D65) luminous flux, $\Phi^{D65}_{v,\alpha}$. However, any other
